# HEALTH PROMOTION FOR PEOPLE WITH EARLY-STAGE DEMENTIA: A QUASI-EXPERIMENTAL STUDY

**DOI:** 10.1093/geroni/igz038.3426

**Published:** 2019-11-08

**Authors:** Ingelin Testad, Martine Kajander, Anne Torsvik Henriksen, Målfrid Meling, Vigdis Vagle, Martha Therese Gjestsen

**Affiliations:** Centre for Age-related Medicine - SESAM, Stavanger University Hospital, Stavanger, Norway

## Abstract

With the limited advancements in medical treatment, there is a growing emphasis on supporting people with early-stage dementia adjusting to their diagnosis and improve their quality of life. This study aimed to evaluate the effects and experiences of people with early-stage dementia attending a 12-week Health Promotion, consisting of 2-hour sessions at weekly intervals focusing on understanding the progression of dementia, promoting physical activity, nutrition, coping, relationships, home- and travel safety, medication and communication with health care providers. Quantitative and qualitative assessments were conducted at baseline and follow-up 1-2 months post intervention, including cognition, self-rated health and depressive symptoms and individual short interviews. A total of 90 persons with dementia participated in this study. The results demonstrated a stable cognitive function, measured by Mini Mental State Examination, of people with dementia during the 4 month follow-up, and a significant improvement in self-rated health measured by EQ VAS (95% CI 0.2 to 7.6, p=0.037). Depressive symptoms measured by Cornell Scale for Depression in Dementia, demonstrated a decline by one point, which is an improvement in depressive symptoms, although not significant. 32 of the 90 qualitative interviews with participants and their carers were included and analyzed with systematic text condensation. The results demonstrated that the intervention was well received by the participants, learning about dementia, practical strategies and focusing on remaining resources was particularly highlighted as well as improved family communication. In conclusion, the intervention had beneficial effects on the cognitive function, self-rated health and depressive symptoms of people with dementia.

